# The Application of Flexible Areas of Interest to Pilot Mobile Eye Tracking

**DOI:** 10.3390/s20040986

**Published:** 2020-02-12

**Authors:** Zbigniew Gomolka, Damian Kordos, Ewa Zeslawska

**Affiliations:** 1 Institute of Computer Science, University of Rzeszow, 35-959 Rzeszow, Poland; 2 Faculty of Mechanical Engineering and Aeronautics, Rzeszow University of Technology, 35-959 Rzeszow, Poland; d.kordos@prz.edu.pl; 3 Department of Information Systems Applications, University of Information Technology and Management in Rzeszow, 35-225 Rzeszow, Poland; ezeslawska@wsiz.rzeszow.pl

**Keywords:** eye tracking, pilot attention, Tobii Glasses Pro, cockpit instruments, object tracking

## Abstract

Recent progress in the development of mobile Eye Tracking (ET) systems shows that there is a demand for modern flexible solutions that would allow for dynamic tracking of objects in the video stream. The paper describes a newly developed tool for work with ET glasses, and its advantages are outlined with the example of a pilot study. A flight task is performed on the FNTP II MCC simulator, and the pilots are equipped with the Mobile Tobii Glasses. The proposed Smart Trainer tool performs dynamic object tracking in a registered video stream, allowing for an interactive definition of Area of Interest (AOI) with blurred contours for the individual cockpit instruments and for the construction of corresponding histograms of pilot attention. The studies are carried out on a group of experienced pilots with a professional pilot CPL(A) license with instrumental flight (Instrument Rating (IR)) certification and a group of pilots without instrumental training. The experimental section shows the differences in the perception of the flight process between two distinct groups of pilots with varying levels in flight training for the ATPL(A) line pilot license. The proposed Smart Trainer tool might be exploited in order to assess and improve the process of training operators of advanced systems with human machine interfaces.

## 1. Introduction

At present, there is a lack of objective systems that would allow for the determination of the level of training of persons operating (not only) mechanical equipment [1,2,3,4,5]. An interesting example is the system examining pilots’ scanning behavior when executing maneuvers at airports in varying visibility conditions [6,7,8]. Furthermore, certain areas of possible application in advanced driver assistance systems and driver training programs have also been proposed [9,10]. The research shown in this paper is a direct response to market demand, because the existing software used in eye tracking examinations performed today utilizing mobile eye trackers does not provide continuous analysis of AOI in the registered video streams. The demand for continuous AOI analysis is great [11,12,13,14,15,16]. The technology shown in the paper will be applied mainly in the industries of aeronautics, forwarding, goods haulage, transportation, and training; however, it is not limited to these alone. The potential groups of receivers of the proposed technology may include: aeronautical and drivers’ training centers, manufacturers of unmanned aeronautical vehicles, and systems that use a Human–Machine Interface (HMI). The potential of the proposed solution is much greater, because it is possible to apply it in the design of diverse interfaces, not only graphical, but also mechanical arrangements. This functionality is crucial especially in the case of human-machine interfaces, where the necessity to transfer vast quantities of data in the shortest time possible is a must, e.g., operators of production lines or operators of terrestrial control stations for systems of unmanned flights [17,18,19,20,21]. Research related to the tracking of pilot’s attention during the most crucial procedures, i.e., take-off and landing, carried out so far had been performed using the SMI RED 500 and Tobii T60 stationary eye trackers and Tobii Glasses mobile eye tracker. The captured video streams were analyzed by stationary eye trackers using environments provided by SMI –BeGaze and Tobii Studio. As an example, the work enabling statistical elaboration of observers attention for the three minute video sequence (MJPG2000, 640 × 480 pixels at 30 fps) including nine instruments in a cockpit requires the effort of a few days of manpower (ca. 48 h of work) [3,22,23]. The effect of this work is non-recurrent and requires redefinition of AOIs both when changing an operator and the registered aeronautical task. The designed Smart Trainer intelligent technology enables both an intelligent localization of AOI during the whole video sequence and acquisition of corresponding observation histograms for individual AOIs defined for the given HMI. The systems determining the training level used currently may be additionally equipped with the proposed solution in order to increase the objectivity and precision in determining the level of achieved skill with a limited input of time.

## 2. Test Stand Used in Eye Tracking Research

Sight is the dominant sense in a human being. It delivers precise information while being an exact and fast stimulus, which activates retina receptors. With the present progress of technology and digitalization, it is possible to analyze focal attention precisely through monitoring of sight functions [24,25,26,27,28]. Non-invasive eye tracking methods contribute to the creation of special optimized glass cockpits, which allow the information to be illustrated on flight parameters in specially created areas in the cockpit, thus committing to their easy perception. The dynamic development of the aeronautical branch forces designers to enable the easiest and fastest delivery of the flight parameter data to the pilot [29,30,31,32,33,34]. Thanks to eye tracking research, it is possible to analyze the distribution of saccades and fixations among pilots with varying levels of flight training. In the experimental part of this research, the Tobii Glasses Eye Tracker system, which is a non-invasive eye tracker allowing for comfortable and fast attention testing without the necessity of immobilizing test subjects’ head, was used. The Tobii glasses employ a mobile eye tracking system utilizing IR (infrared) markers to define the Areas Of Analysis (AOA), which enable automatic data aggregation. AOAs enable the acquisition of visualizations and statistics from the gathered eye tracking data.

**Figure 1:**
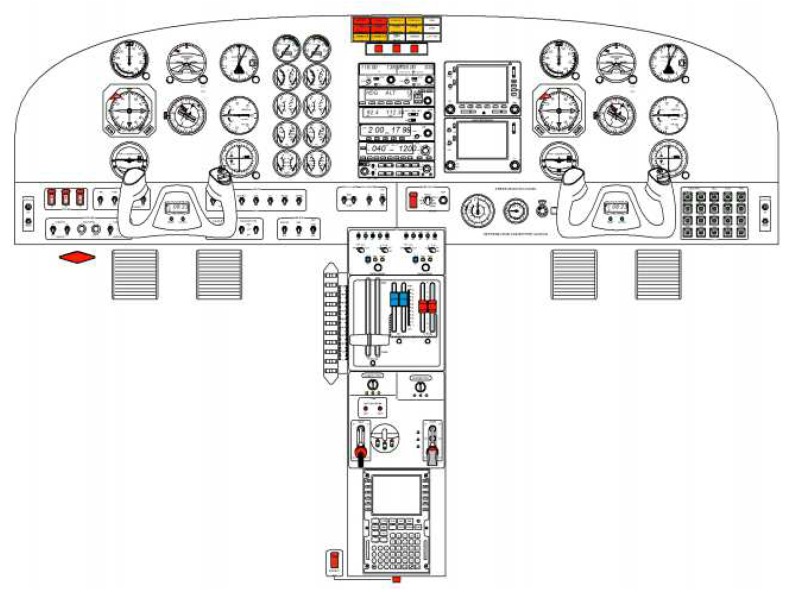
FNTP II MCC simulator: twin engine piston aircraft with folding landing gear [35].

In order to ensure experimental conditions representing a real plane flight scenario as closely as possible, a professional flight simulator used for flight training was used. The FNTP II MCC simulator (Figure 1) is an alternative for pilots training practically in the air. Thanks to the possibility of operation in diversified modes, it allows the emulation of the real-life behavior of a plane and the systems placed in its cockpit. With its help, it is possible to configure, monitor, assess, and save the training tasks for future analysis.

For the needs of the experiment, the simulator was configured as a twin engine piston aircraft with folding landing gear with analogue equipment. The aircraft’s configuration corresponded to the final landing approach, i.e., flaps in landing position, landing gear lowered, and power output close to minimum. The communication and navigation frequency set with the flight according to the Instrument Landing System (ILS) with lateral and vertical guidance would be possible. For the cockpit organized in such a way, it was possible to carry out the attention trajectory measurements.

## 3. Test Stand Description and the Assumed Air Task

The experimental part of the research was carried out with the Department of Avionics and Control Systems of Rzeszow University of Technology. The research was carried out in cooperation with the students of the Flight Training Center enrolled in a diploma course training for the Commercial Pilot License CPL(A). The presented research is a continuation of the research project related to the examination of sight activity of a pilot during the flight. The analysis and visualization of the registered pilot’s eye attention using the SMI RED500 stationary eye tracker was described in [3,22]. During the tests performed on the simulator, the following assumptions were introduced:

- The test stand was comprised of a mobile eye tracker in the form of glasses, a control wheel, and rudder pedals and the stereoscopic image from the projectors.
- During the individual session in the progress of the experiment, conditions, which limit external environment influences (excessive lightning, noise), were introduced.
- The tests were performed in the Seneca II aircraft equipped with analogue instruments placed in the front part of the cockpit allowing for navigation in the visibility conditions.
- The aircraft pilotage was possible thanks to the use of steering only through the throttle, control wheel, and rudder pedals.
- The plane in which the flight was performed had a full landing configuration and was adapted to perform the requested part of the flight.
- The experiment lasted no more than 30 min in order to improve the pilot’s perception and limit the influence of fatigue. It allowed for better focus of attention.
- The group of people with flight training for the instruments’ indication, having the Instrument Rating (IR) license, was acknowledged as an Instrument Flight Rules (IFR) pilot, whereas the group that did not have these licenses was acknowledged as a Visual Flight Rules (VFR) pilot.
- The analysis of the obtained results was carried out using the Tobii Pro Studio software and the Smart Trainer application designed for the analysis of saccade and fixation distribution in time.

**Figure 2:**
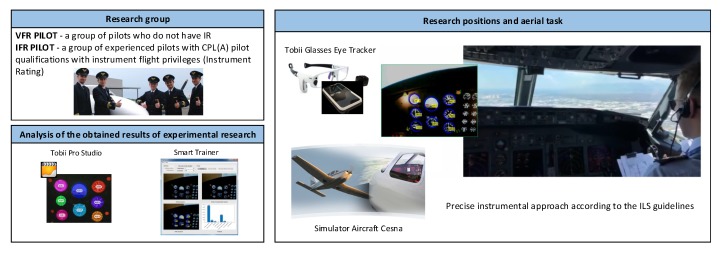
Experiment procedure scheme.

Figure 2 shows the diagram of the performed experiments.

**Figure 3:**
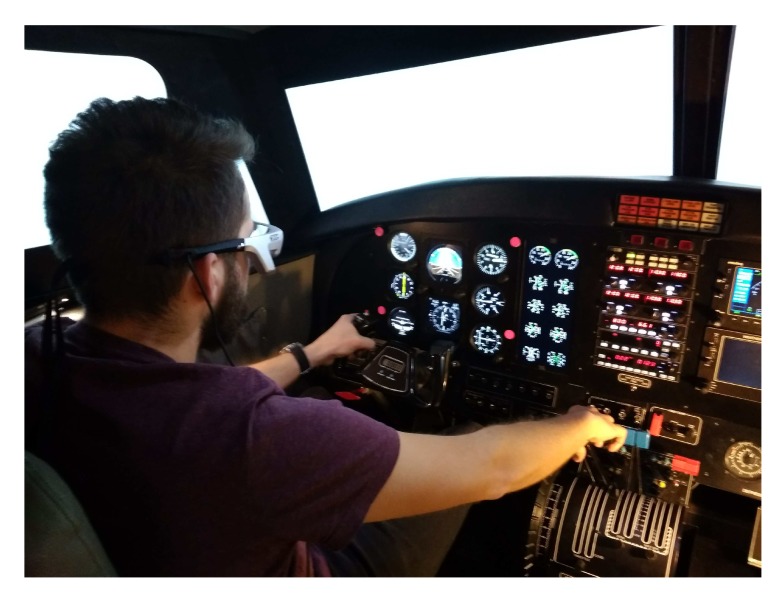
Test stand, with introduced markers, used for the pilot’s attention examination.

The experiments were performed with the aid of the Tobii Eye Glasses mobile eye tracker by Tobii, equipped with the camera to register left-eye pupil position. The Tobii Pro Studio programming environment and a set of infrared markers allowed the stand to be calibrated, recorded in the register, and perform readout and analysis of the obtained data. The simulator used for the research was an FNTP II MCC with a twin engine piston aircraft with folding landing gear. The steering with the control surface was performed using the control wheel, which corresponded to the bank and pitch of the aircraft. The rudder pedals controlled the yaw of the aircraft. Additionally, in order to facilitate analysis, in the key area due to the performed flight task, four red markers that stood out in the observed area were introduced. The stand used for research together with the introduced markers is shown in Figure 3.

### The Characteristics of the Distinguished Test Subjects Groups and Their Task

During the candidate selection for the research, the main factor was the completed flight training for the Instrument Flight Rules (IFR). Such a license allowed the pilots to perform flights under weather conditions characterized by low visibility (below 5 km) and cloud base making visual terrain reference impossible. At the same time, two groups were formed, based on the flight experience:

- IFR pilot: for the pilots with an IR license,
- VFR pilot: for the pilots with basic training for the flights with visibility.

Five persons were assigned to each group. The specification of the groups is shown in Table 1.

**Figure 4:**
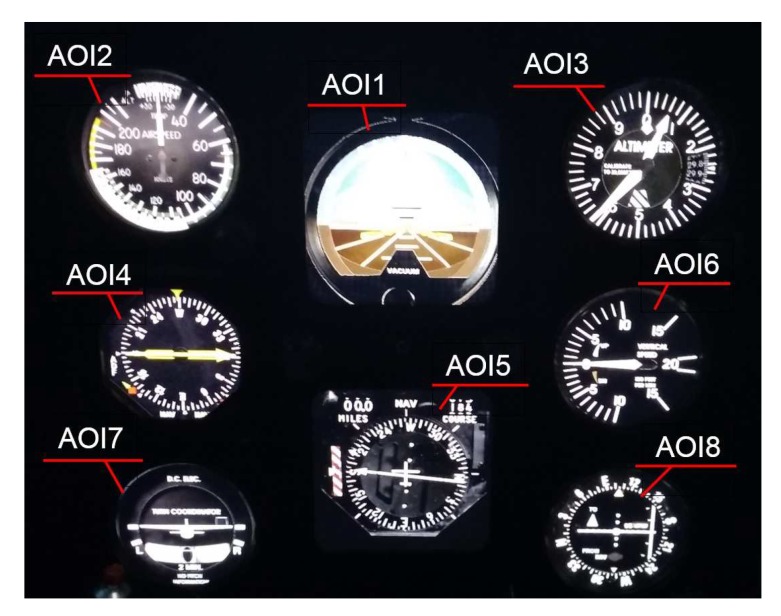
The view of the cockpit instruments used in the aircraft model employed in the experiment.

The pilots who took part in the experiment were asked to perform one task. It comprised the execution of a precise instrumental approach according to the Instrument Landing System (ILS). Such an approach ensured vertical and lateral guidance to the Decision Altitude (DA). The barometric height of DA was referenced to the local pressure at average sea level: local pressure (QNH). The approach was performed with minimal weather conditions for CAT I ILS, which is RVR visibility along the start path of 550 m, cloud base at 200 ft Above Ground Level (AGL). The atmospheric conditions allowed only for IFR flight, which caused the pilots to be able to navigate only using cockpit instruments. The flights were performed at EPRZ-Rzeszow Jasionka airport, on Runway No. 27, with a landing length of 3192 m and a breadth of 45 m. The airport elevation was equal to 693ft, and the magnetic heading of the runway was $265^{\circ }$. The pilots started the flight 7NM away from the runway threshold at the barometric altitude of 3000 ft Above Medium Sea Level (AMSL). The task ended upon reaching a Decision Height (DH) of 200 ft. The instruments that were at participants’ disposal during the flight task’s execution in the experiment are shown in Figure 4.

The individual instruments used in the research are described below:

- AOI1: Attitude indicator (artificial horizon); supplies information about aircraft position in space,
- AOI2: IAS (Indicated Airspeed Indicator); indicates the instrumental speed of an aircraft relative to the air,
- AOI3: Altimeter; informs about the barometric altitude above the mean sea level,
- AOI4: RMI Indicator (Radio Magnetic Indicator); informs about the position of the aircraft relative to the Non-Directional Beacon (NDB);
- AOI5: VOR/ILS indicator (Very high-frequency Omnidirectional Range/Instrument Landing System); ensures the vertical and lateral guidance during an ILS approach,
- AOI6: Vertical speed indicator; informs about the rate of climb or decline of an aircraft,
- AOI7: Turn coordinator; assists in the performance of a coordinated turn with constant angular velocity,
- AOI8: CDI (Course Deviation Indicator); is an alternative source of indications of VOR/ILS.

## 4. Methods

**Figure 5:**
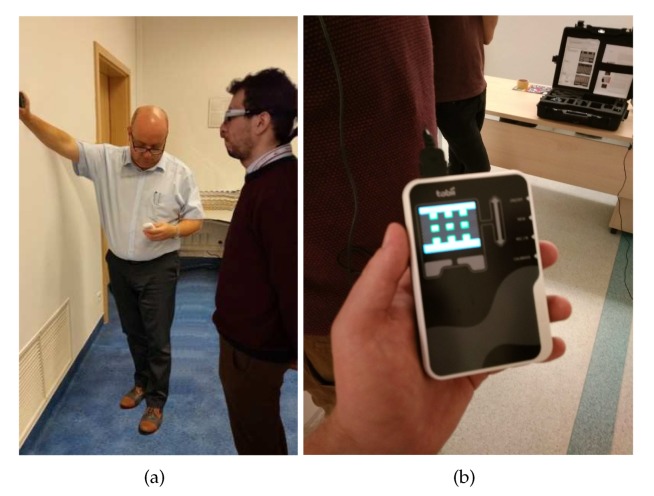
(**a**) Calibration with the IR marker; (**b**) precise calibration, no repetition necessary.

After of group of pilots to participate in the experiment was selected, the first stage of experiment, which was calibration of the Tobii Pro Glasses eye tracking glasses, was started. In order to obtain accurate data from the video recorder, a nine point calibration was used, which allowed the precise assessment of the usability of the performed sessions. The examined subject was placed at a distance corresponding to the relative distance between the pilot’s head and instruments panel on the flat surface background (see Figure 5a). Nine calibration points were initialized using the IR marker on its background. The recording assistant enabled real-time calibration monitoring during the process. This way, the precise positioning of the IR marker relative to the glasses was enabled. The calibration was finished by lighting up the nine calibration points with green illumination (see Figure 5b), which allowed progress to the next stage of examination, which was the flight task.

After the device was calibrated, the pilot assumed the position at the control wheel and performed a flight, which was registered with the glasses. The data registered with Tobii Glasses Pro and saved on the SD Recording Assistant allowed the generation of files with Tobii Pro Studio specific extension. For the need of the analysis of data obtained from the sessions, the dedicated software was used to generate files for videos registered by the recording camera located in the pilot’s glasses. The videos were recorded in 640 × 480 pixels resolution at 30 fps. The Tobii Pro Studio software allowed exporting data related to the pilot’s eye attention trajectory during the registered test sessions into *.xls format, which was compatible with the MATLAB environment.

**Figure 6:**
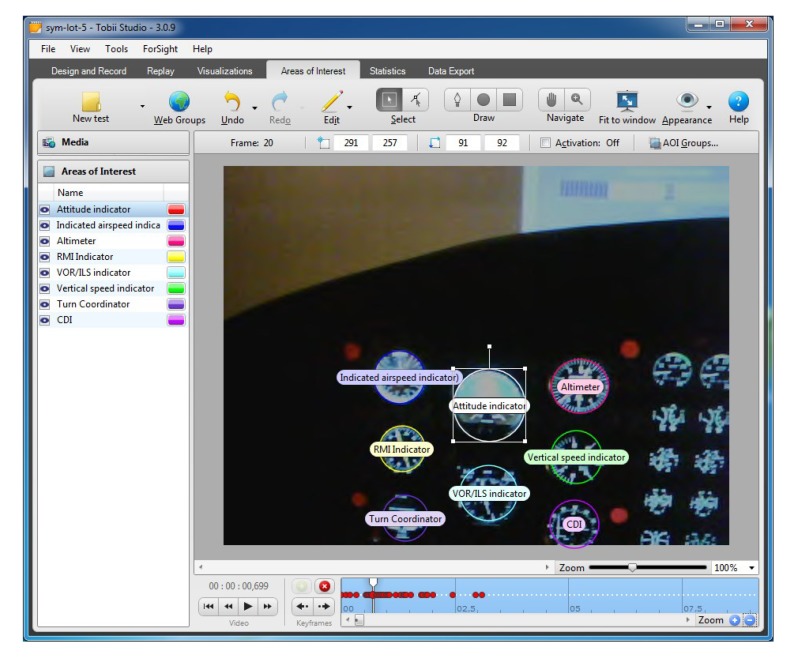
Tobii Pro Studio software used in order to define AOI corresponding to the positions of cockpit instruments.

Tobii Pro Studio as a platform dedicated to the analysis of the performed experiment employed an option to overlay the so-called AOI, which enabled the creation of defined areas of interest in order to analyze a number of fixations in the given area (Figure 6). When the observed scene changed, the positions of individual instruments relative to the static AOI changed according to the movement of the pilot’s head. As a result, there was a necessity to redefine the coordinates of AOIs at each key frame, which is denoted as small red circles at the bottom time axis.

**Figure 7:**
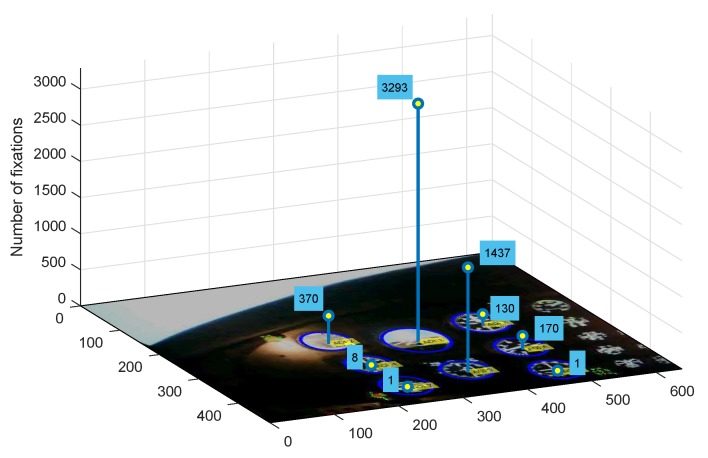
The number of fixations formed on the basis of AOI analysis by Tobii Pro Studio software with individual analysis of particular instruments.

According to the dynamics of the observed scene, this amount of key frames may increase. Despite this, in order to conduct further analysis, it was necessary to modify the AOI position manually for each subsequent frame in the video sequence (post-processing mode). The result of that was an increase in the computational complexity and a very long refresh time of the Tobii system interface. Most importantly, such an obtained trajectory of AOIs in a given project could not be applied to new unseen video streams. This fact was the main motive to create a tool that would allow for the analysis of the stream recorded with Mobile Tobii glasses. The designed project and exemplary video analysis in the Tobii Pro Studio environment was performed on a premium class computer with the following parameters: Intel Core i5-4330 M CPU @ 2.80.GHz 2.80 GHz processor, 16 GB RAM, graphics cards: NVIDIA GeForce GT 730 M, Intel HD Graphic 4600, system: Windows 10. AOI objects introduced in the consecutive frames considerably increased the complexity of vector graphics of individual AOI included in the analyzed video stream. As a consequence, the Tobii system refresh time increased up to a few minutes per single video frame, making it impossible to further edit the project. This method proved highly inefficient, because the analyzed video, which lasted 5 min 29 s, comprised 9870 frames, which made precise analysis impossible. Based on these observations, the authors later proposed a system Smart Trainer for dynamic AOI tracking and analysis in the video stream. Preliminary observation results obtained in this way for the manually designed AOI motion path were used to assess the effectiveness of the Smart Trainer tool on the same exemplary video sequence. Compliance of histograms obtained from the Tobii Studio system and Smart Trainer on the same input data confirmed the main assumptions of the technology being developed and the effectiveness of its operation. Based on this observation, all other video sequences in the experimental part of the studies were processed exclusively using the Smart Trainer. As a result of the application of an algorithm for overwriting AOI position in the consecutive frames of the video and performing individual analysis for each instrument separately, the number of fixations for one video sample was obtained (Figure 7).

The direction of causality employed to obtain the above analysis prompted an idea about the creation of a more efficient method of examination of the obtained results. Tobii Pro Studio software enabled the export of tabulated data including information about the number of fixations and their coordinates. For each video sample, the data were exported to an .xls file, which allowed for their further analysis with the MATLAB software. In the obtained data, the information about the current video resolution, frame capture speed, position, and duration of observer’s attention was used for further analysis of the attention:

- GazeEventDuration, duration of gaze,
- FixationPointX (MCSpx), X coordinate of a fixation,
- FixationPointY (MCSpx), Y coordinate of a fixation.

Considering the limitations of shared analysis of multiple AOI concurrently with the high dynamics of the changes of the observed scene in the Tobii system, the Smart Trainer application in MATLAB environment was designed. An assumption was made that the following functions would be included in the designed application:

- It would enable concurrent reading of the saved video stream in avi format, conforming to the codecs set provided by MATLAB and the xls dataset including the fixation times and coordinates,
- It would allow the definition of markers, which would facilitate tracking of the coordinates of the simulator’s cockpit,
- It would allow for interactive definition of coordinates and sizes of individual AOI for the selected instruments,
- It would comprise the implemented marker tracking mechanism for the markers placed in the pilot’s field of view,
- It would allow the definition of transformation parameters for the moving components of the scene, which would allow definite changing of AOI coordinates for particular instruments,
- It would allow for parametrized blur of contour outlines defining the individual AOI, for example using the Butterworth filter profile in 2D scenery,
- It would display the video stream containing the defined AOIs and their changing position for the individual video frames,
- It would allow for the visualization of particular stages of analysis of the chosen video sequences taking into account their varying duration time,
- It would enable attention histogram construction for the set of analyzed video streams,
- It would allow the export of the obtained results to xls format for further processing and interpretation compatible with the needs of this research.

**Figure 8:**
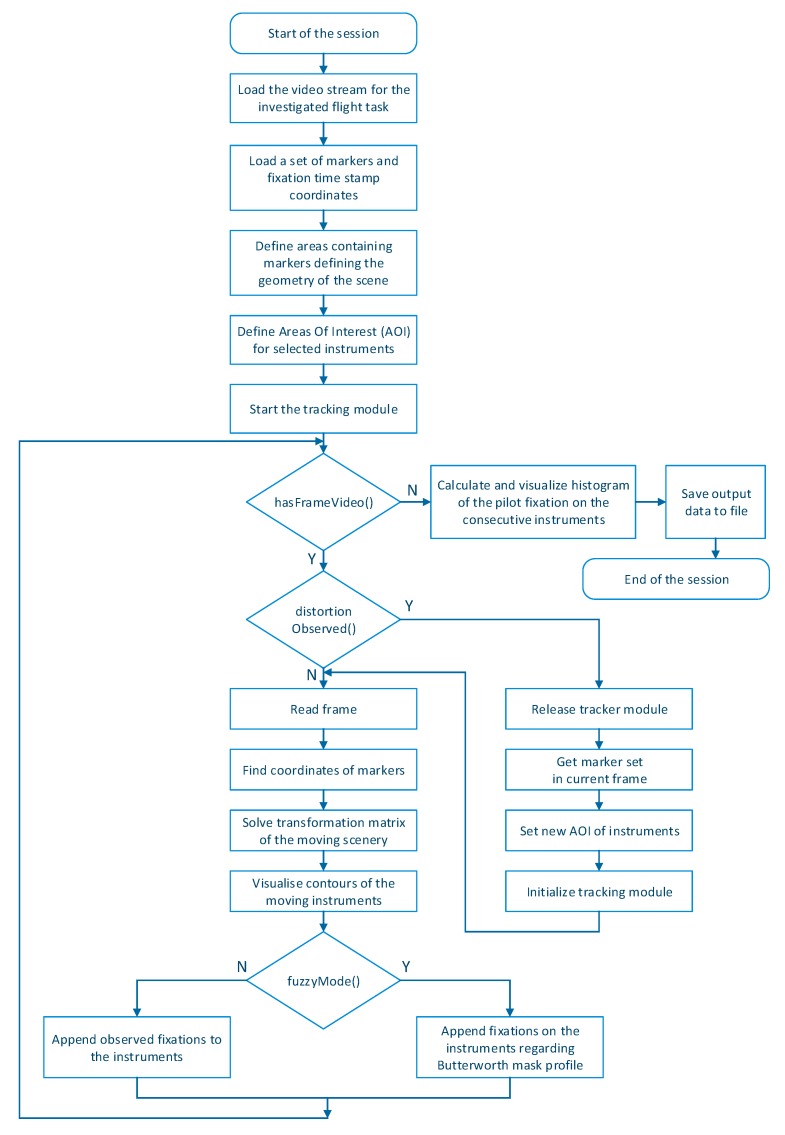
The operation scheme of the designed algorithm.

**Figure 9:**
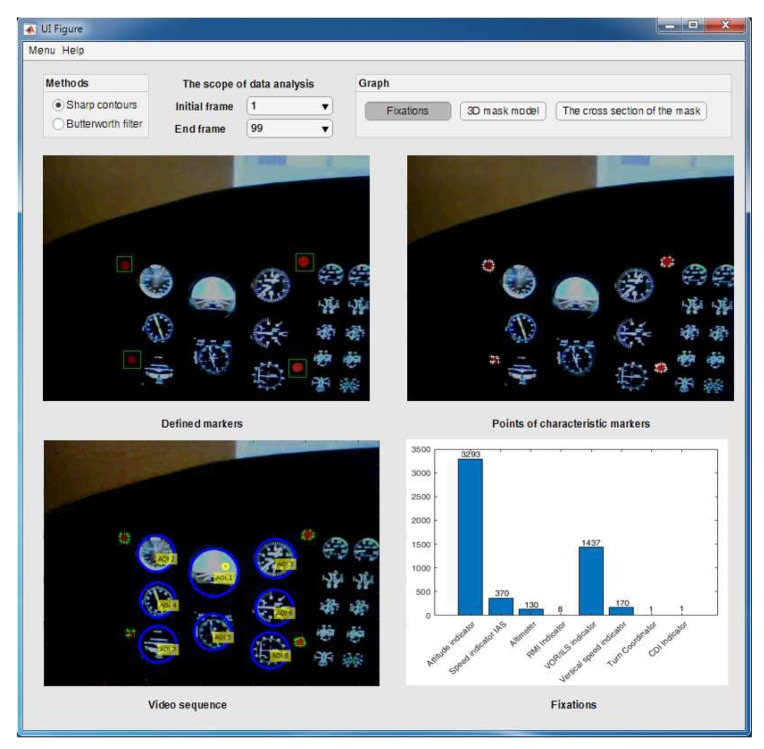
Smart Trainer application interface.

Considering the requirements formulated above, the Smart Trainer application, which allowed for dynamic calculation of fixations in the particular areas of pilots’ attention during the performed flight task, was designed. This permitted the creation of a universal and advanced algorithm for tracking of selected objects in the scenery observed by a pilot. The operation scheme of the designed algorithm is shown in Figure 8, and the view of Smart Trainer application is shown in Figure 9.

## 5. Results

The Smart Trainer was used to perform an analysis of video material, which was used during work with an AOI set defined analogically in Tobii Pro Studio software. The comparison of the results obtained with the Smart Trainer and the analysis obtained with Tobii Pro Studio are shown in Table 2.

Assuming that the values obtained while using Tobii software were a reference point for comparative analysis of the results shown above, the statistical analysis was employed to assess the usability of the Smart Trainer application. Absolute and relative errors were determined for the defined video material. The number of registered fixations divided by the area of their occurrence is shown in Table 3.

The results above enabled analysis of the absolute and relative errors for the two methods of determining a number of fixations. The value of relative error was also determined taking into consideration the percentage contribution of the number of fixations in the whole test, which served as a decisive factor. The values of errors: absolute, relative, and relative taking into account the weight of an instrument for every instrument are listed in Table 4.

It was noted that the relative error taking into account the percentage number of fixations at given AOI made it possible to state that the process of determining the number of fixations utilizing the Smart Trainer was very precise and thus allowed for further data analysis with it. It was also noted that the analysis of the performed experiment that utilized Tobii Pro Studio software was ineffective and incorrect in relation to the tests carried out. The authors decided that the designed application would enable effective and adequate analysis of fixations in the AOI and would be utilized for the analysis of the registered video sequences.

Two groups of test subjects were specified in the performed tests: the VFR pilot and IFR pilot. The time frame, in which the analysis based on AOI was performed, was adjusted for individual pilots. Thus, information about the number of fixations in given areas was obtained. Every flight was completed by every pilot with varying execution times. The Smart Trainer was used to analyze the video sequences obtained for individual groups of pilots. Due to the varying duration of individual flights, varying numbers of fixations were registered. The number of fixations registered for individual pilots from the VFR pilot and IFR pilot groups is shown in Table 5.

The following research was related to the determination of the fixations’ distribution in the defined AOI, and the obtained results are shown in Table 6. The obtained research results allowed the conclusion to be drawn that attention in the VFR pilot group was directed mainly at the attitude indicator, which informed about the position in space of an aircraft, and at the VOR/ILS indicator, which ensured lateral and vertical guidance during the approach in ILS. In the IFR pilot group, it was observed that the attention of examined test subjects was first directed at the attitude indicator, then at the VOR/ILS indicator and at the vertical speed indicator, which informed about the climb or descent rate of an aircraft. The results obtained for both groups indicated that pilots’ training and their reaction to individual instruments were correct for the performed task.

Due to the varying duration of task execution, the percentage the contribution of fixations in the examination was calculated for the obtained values. This allowed the comparison of the contribution of the number of fixations in the individual AOI to the number of all fixations in AOIs. The obtained results are shown in Table 7.

### 5.1. Gradation of Attention for IFR and VFR Pilot Groups

**Figure 10:**
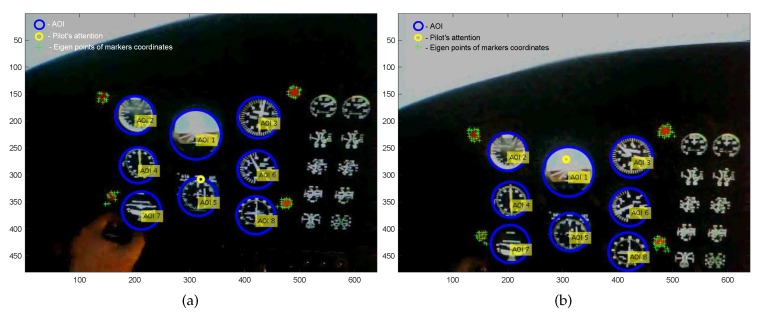
An example of the not satisfied (**a**) and satisfied (**b**) condition of the containment of pilots’ eye attention within AOI.

The results obtained for all groups of pilots allowed the formulation of the conclusion that a large part of fixations was located outside the boundary area of individual AOI and thus encouraged reflection on the validity of the formulated conclusions. The example positions of the pilots eye’s attention are shown in Figure 10.

In the case when the condition of the containment of pilot’s eye attention within AOI was not fulfilled (see Figure 10a), it may be acknowledged that the applied condition of containment within AOI was not satisfied, because the coordinates of attention were not contained within the unequivocally defined circle area. The use of Tobii glasses, which were subject to uncontrolled pilot facial movements and complex calibration, which ensured the measurement with assumed accuracy, allowed it to be stated that the attention depicted in Figure 10a permitted the conclusion that an instrument was being observed despite the unequivocal condition of containment in the closest AOI not being fulfilled. Therefore, it was possible to state that with external interferences, movements performed by a pilot, or hardware configuration, which fulfilled the minimal calibration requirements, the majority of fixations were omitted. Thus, it was concluded that the applied condition of containment within a circle may be switched to another one, which would take into account an additional coordinate axis and would bring the imaging function coordinate system to 3D. Application of a 3D mask for instruments observed by a pilot was possible through the use of a modified Butterworth filter. The classical form of the Butterworth filter is defined by dependence [36]:

(1) $$H\left(u,\right.v)=\frac{1.0}{1.0+c\left[D\left(u,\right.v)/D_{0}\right.{]}^{2}}$$

where $D_{0}$ represents the half value of the filter. For the needs of the simulation tests, the modified form of the filter, which would constitute the 3D model of the individual instruments AOI’s defining mask, was used. Assuming *x* and *y* as the coordinates of the imaging function, it was assumed accordingly:

(2) $$Mask\left(x,\right.y)=\frac{1.0}{1.0+c\left[\left(\left(x-\right.x_{0}{{)}^{2}-\left(y-\right.y_{0})}^{2}/r_{0}\right)\right.{]}^{n}}$$

where $x_{0}$, $y_{0}$ are the coordinates of the mask’s center, $r_{0}$ the radius of the instrument’s mask, *n* the degree of the modified Butterworth filter, and c=0.414 the constant of a filter, for which the profile of the instrument’s mask achieved the so-called half point.

**Figure 11:**
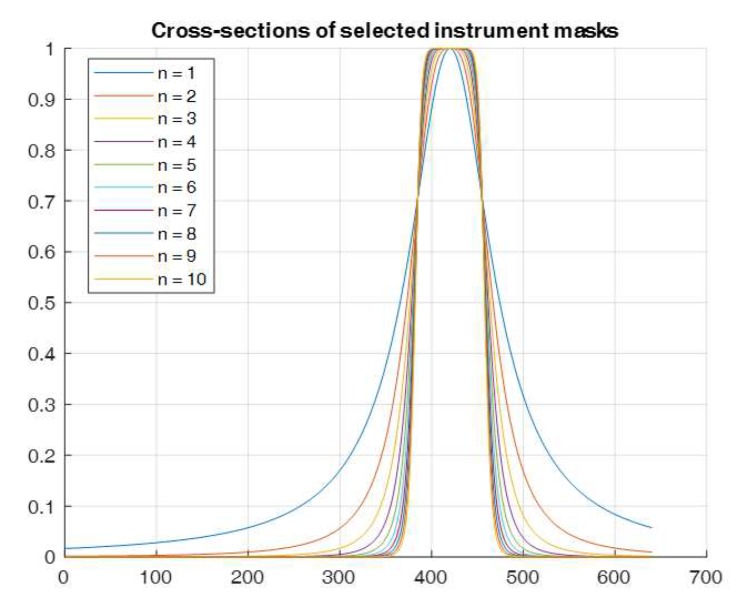
The chosen instrument masks used for the constant radius with decreasing mask profile *n* from 1, sharp, to 10, smooth.

The example progressions for the instrument masks obtained with the above Equation (2) are shown in Figure 11. The degree *n* of the instrument mask’s profile varied in the range $n\in \left(1,10\right)$.

**Figure 12:**
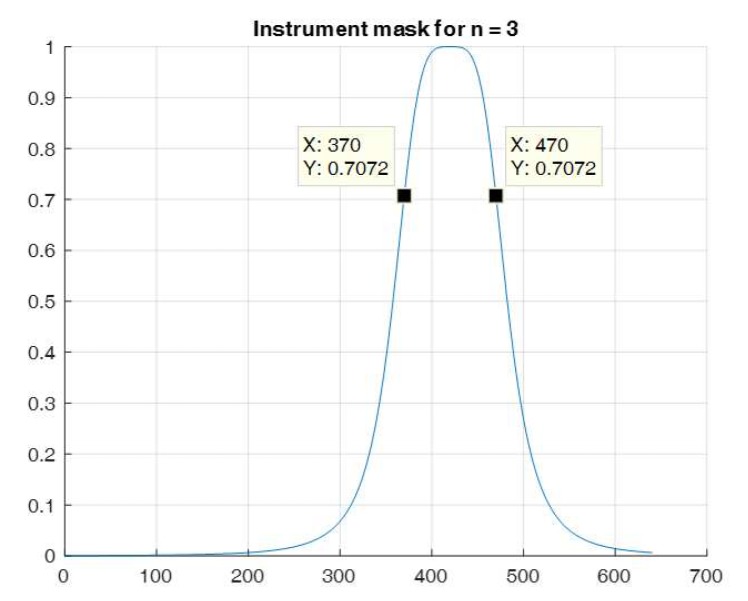
Section of single instrument’s mask for n=3, r=50.

In Figure 12 the section of single instrument’s mask is shown for the chosen parameters. Assuming such a model for the construction of individual AOI, it was possible to state that their blurred contours may come into mutual coincidence.

**Figure 13:**
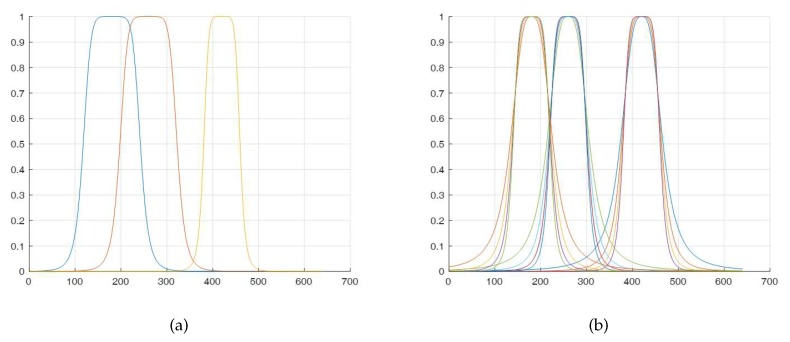
Possible forms of large (**a**) and small (**b**) coincidence of individual AOIs respectively.

By assuming a relatively high coefficient of outline blur, a situation, where individual AOI may unintentionally register attention in parallel, may be caused. Figure 13 shows an example of such a situation.

**Figure 14:**
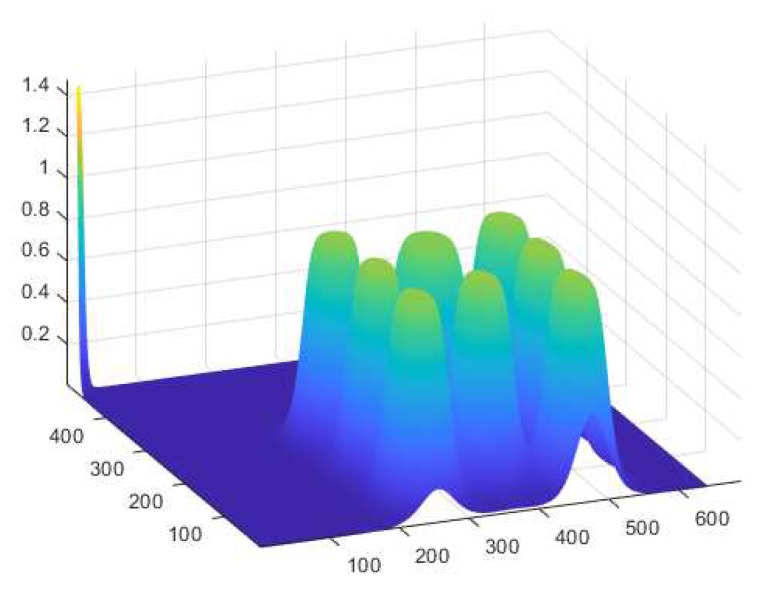
3D mask of the observed area of instruments.

Taking the above-mentioned phenomena into consideration, for the experimental part of the research, the instrument’s outline profile selection was performed in a way that minimized the effect of multiple registrations of attention into several AOIs at the same time in a given moment of observation. Moreover, it may be assumed that the properly smoothed form of instrument mask minimized the effect of coincidence of individual AOI, and an error introduced potentially for the typical observation trajectory did not influence the final results of pilot’s attention registration in a significant way. The results of the tests of the comparison of attention registration for the same piece of observation performed in the Tobii environment and the analysis executed in the Smart Trainer application proved the above conclusion. The following stage of the tests was connected with the visualization of the obtained 2D model into 3D coordinates. The adequate selection of mask parameters and elimination of the coincidence effect allowed creating a final version of the mask for the panel of observed instruments. In Figure 14, the instrument mask for pilot’s attention located outside the observed area is shown. This may represent a situation when one of the observer’s eyes is temporarily closed or the sight is directed outside of the designated observation area.

**Figure 15:**
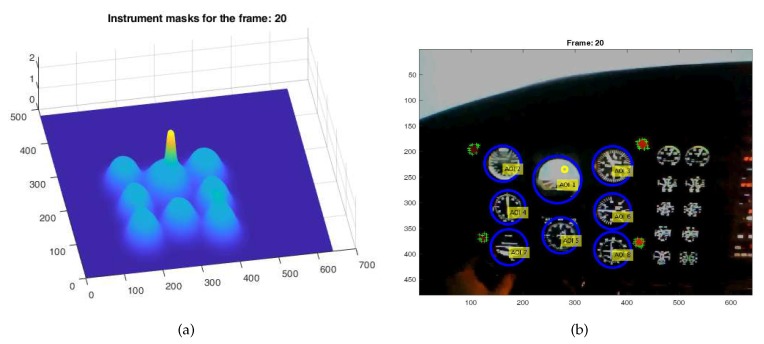
Instrument masks’ outlines position (**a**) and observers’ attention for Frame 20 (**b**).

An example of the observed picture frame for the 3D coordinate system for the chosen picture frame is shown in Figure 15.

The selection of the blurred function based on the Butterworth filter profile ensured exact registration of occurring fixations of pilot’s attention. It has to be noted that the use of such algorithm not only increased the accuracy of the performed analysis, but also increased the accuracy of the measurement itself, ensuring a more precise result of the eye’s attention registration through Tobii Glasses. Undesirable glasses movements in relation to the pilot’s nose may adversely influence the measurement.

### 5.2. Comparative Analysis of the Results

**Figure 16:**
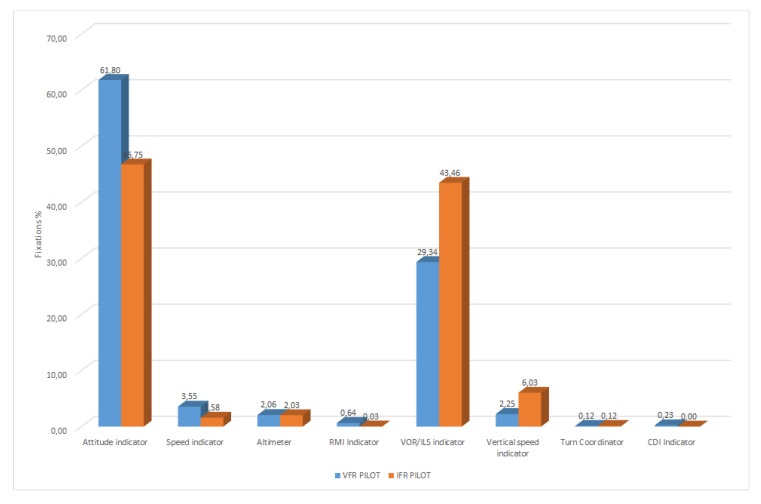
Average percentage time contribution for the two examined groups.

After considering the variable experiment duration periods, the obtained results were averaged for ten examined persons, and the percentage contribution of individual instruments in the process of cockpit observation (see Table 7) was obtained. The analysis of the average percentage time contribution of fixation, which allowed determining the essential instruments for the persons belonging to the VFR pilot and IFR pilot groups, was done (see Figure 16).

The obtained results showed that for the VFR pilot group, the most crucial AOIs (above 2%) were:

- Attitude indicator: 61.80% of average test time,
- VOR/ILS indicator: 29.34% of average test time,
- Speed indicator: 3.55% of average test time,
- Vertical speed indicator: 2.25% of average test time,
- Altimeter: 2.06% of average test time.

Whereas for the IFR pilot group, the most crucial AOIs (above 2%) were:

- Attitude indicator: 46.75% of averaged test time,
- VOR/ILS indicator: 43.46% of averaged test time,
- Vertical speed indicator: 6.03% of averaged test time,
- Altimeter: 2.03% of averaged test time.

After application of the diffused AOI outlines method, the percentage contributions for the two methods used for counting fixations in the video stream, i.e., sharp outlines and utilization of blurred function, were obtained. The obtained results are shown in Table 8 and Table 9.

The results obtained above allowed it to be stated that for the characteristics of the performed task, the method employing gradation of pilot’s attention enabled the acquisition of a greater number of fixations within the characteristic areas. For the task focused on the execution of flight in no visibility conditions and based on reference only to the cockpit instrument, this method was highly effective, showing a small number of fixations outside AOI. By averaging the percentage results from Figure 16, it was shown that during the average duration time of the test equal to four minutes 29 s, exactly 87.04% of fixations were located on the defined AOI. Therefore, it may be concluded that in the remaining 12.96% of the whole experiment’s duration time:

- Saccades took place.
- Blinking took place, which caused a loss of the eye’s XY coordinates.
- The fixations were located outside AOI.

Having the research results at our disposal, a summary was created (see Table 10), which compared attention focus for the two groups of examined pilots obtained using the algorithm that employed attention gradation. These results were averaged, and the average percentage values of pilots’ attention in the VFR pilot and IFR pilot groups were obtained (Table 11).

Based on the obtained results, the following conclusions may be formulated. The pilots attention in both groups was directed mainly at the attitude indicator. For the flights without visual reference to the terrain, this instrument plays a crucial role, due to the maintenance of appropriate angles of spatial orientation in order to secure a controlled flight. Supervision of the attitude indicator is desirable in the process of training apprentice pilots. Placing of both IFR and VFR pilots’ eye attention in this area indicates a correct training. It should be noted that for the VFR group, this instrument constituted over a half (52.24%) of all fixations. For the assumed weather conditions and slight speed changes, the constant pitch of an aircraft ensured a stable landing approach. At the same time, it eased maintaining within the glide slope of precision the ILS CAT I approach used in the research task. The second most important instrument, at which both groups of pilots kept their eyes’ attention, was the VOR ILS indicator. The IFR pilot group focused 42.24% of their attention on the area of this instrument, which was 7% more than in the case of the VFR pilot group. In terms of the basic task performed in the IMC conditions, the indications of the VOR ILS indicator were a basic source of information about the correctness of the executed ILS CAT I approach. This instrument also allowed for the determination of deviation from the required vertical and lateral approach path. The rate at which indication of this instrument changes enabled also determining the aircraft movement tendency. The skill to introduce flight trajectory corrections based on the VOR ILS indicator is a characteristic of pilots with the instrument rating license. This was confirmed by the high percentage of fixations on this instrument in the IFR pilot group. A significant discrepancy between the two groups existed in the number of fixations observed in the vertical speed indicator. The vertical speed of an aircraft, which enabled precise guidance along the vertical path of the ILS, constituted 6.01% of all fixations for the IFR pilot group. For the VFR pilot group, it was only 3.20%. The speed indicator turned out to be an instrument on which the non-IR pilots focused more attention than the IR trained pilots. For the meteorological conditions during the performed task assumed in the simulator and for slight changes in the throttle position, ensuring a constant pitch of the aircraft did not influence the change of the aircraft’s airspeed. At the same time, the information about the indicated speed was a superposition of the indications of the vertical speed indicator and attitude indicator. This may indicate low focus of attention on this instrument by the persons belonging to the IFR pilot group. The number of fixations registered in the area of the altimeter for the IFR was close to the number of fixations for the VFR group. In both groups, the altimeter was given attention for ca. 3% of all fixations. Due to the characteristics of the performed task, it only had an auxiliary function in order to determine the point at which the research task was finished. The amount of fixations registered for the remaining instruments was below 1% and proved that they were not the key instruments with respect to the characteristics of the ILS CAT I approach performed. There was no significant difference visible between the representatives of the VRF pilot and IFR pilot groups. From a flight safety point of view, both test groups performed the task correctly. The difference in monitoring of the vertical speed indicator could be noted at the moment of landing approach between the two groups. In the case of an experienced pilot, this occurred more frequently and served to maintain the optimal path of the approach. The unexperienced person monitored this indicator less frequently. After examining both groups of pilots, it could be said that the correct execution of the instrumented ILS CAT I approach in conditions with no visual reference to the terrain was possible with the use of five instruments (attitude indicator, VOR ILS indicator, vertical speed indicator, speed indicator, altimeter). This was an indication of the difficulty level of the presented task and the necessity of the correct readout of multiple instruments in the correct order and with specified frequency. It is worth noting that the precision of task execution was not taken into account in the experiment.

## 6. Conclusions

The experiments presented in this paper were carried out in the area of registering and tracking of key instruments in the cockpit using the mobile Tobii Glasses Pro eye tracker. The main target of the proposed research was to design an intelligent technology that would enable registration and analysis of the attention of operators of mechanical devices, in which human-machine interaction occurred, in order to support the process of training and analysis of the acquired skills. The Smart Trainer technology using an intelligent algorithm, which overcame the limits of eye tracking systems and which allowed for dynamic tracking of selected objects and further analysis of attention trajectory, was developed. The realized research showed the possibility to assess the training level based on the eye tracking system. The research was carried out on a test group of pilots with varying training levels and flight experience, and its results unequivocally indicated the possibility to use the proposed technology for assessment of acquired skills. It was proven that the registered attention analysis tools employing mobile eye trackers available on the market did not provide mechanisms for flexible selection of AOI objects, which could be tracked in the registered video stream. This disabled the further analysis, which was crucial for the assessment of the skills acquired in the process of the training of operators of such systems. It was proven by experiment that by using tracking algorithms and the outline blur mechanism, it was possible to detect the localizations of selected objects in the video stream and, on this basis, to design fixation statistics effectively. In order to develop the proposed intelligent Smart Trainer technology, further research using a flying lab will be performed, which will allow the use of deep neural network, enabling the use of such technology for the analysis of the operator’s attention in the application for various objects (instrument sets). Such a strategy makes the proposed technology universally applicable for various areas. The proposed technology will allow the assessment of the ergonomics of various HMI interfaces, the need to control both widely understood machines, as well as processes, in which the key factor is a fast and precise delivery of the highest amount of information possible to the operator.

## Figures and Tables

**Table 1:** Specification of the groups of persons taking part in the experiment.

Group	No. of Test Subjects	Age	Sex
VFR pilot	P01	24	Male
	P02	24	Male
	P03	23	Male
	P04	23	Male
	P05	23	Male
IFR pilot	P06	25	Man
	P07	25	Male
	P08	24	Male
	P09	24	Male
	P10	26	Male

**Table 2:** The number of fixations registered with Tobii Pro Studio and the Smart Trainer.

Instruments	Tobii Pro Studio	Smart Trainer
Attitude indicator	3293	3110
Speed indicator	370	310
Altimeter	120	112
RMI indicator	8	20
VOR/ILS indicator	1437	1433
Vertical speed indicator	170	116
Turn coordinator	1	0
CDI indicator	1	2

**Table 3:** The number of fixations taking into account the area of their occurrence.

Name	Value
The number of fixation occurring at AOI	5103
The number of fixations occurring outside AOI	2424
Total number of fixations	7527

**Table 4:** Percentage contribution of individual instruments in the process of vision: VFR pilot.

Instruments	Absolute Error	Relative Error (%)	Relative Error Taking into Account Number of Fixations (%)
Attitude indicator	183	5.557243	3.382625
Speed indicator	60	16.216216	1.109057
Altimeter	18	13.846154	0.332717
RMI indicator	12	150.000000	0.221811
VOR/ILS indicator	4	0.278358	0.073937
Vertical speed indicator	54	31.764706	0.998152
Turn coordinator	1	100.000000	0.018484
CDI indicator	1	100.000000	0.018484

**Table 5:** Number of fixations with distinguished area of occurrence.

Pilot Group		Fixations at AOI	Fixations Outside AOI	Total Number of Fixations
VFR pilot	P01	6299	1330	7629
	P02	1644	5431	7075
	P03	5103	2424	7527
	P04	2110	4676	6786
	P05	4522	2378	6900
IFR pilot	P06	5252	1151	6403
	P07	6372	770	7142
	P08	5665	1447	7112
	P09	4094	2555	6649
	P10	6104	1905	8009

**Table 6:** The number of fixations registered by the Smart Trainer for the AOI in the VFR and IFR pilot groups.

Instruments	Value									
	VFR Pilot					IFR Pilot				
	P01	P02	P03	P04	P05	P06	P07	P08	P09	P10
Attitude indicator	4374	532	3110	1751	3030	2230	2489	2250	2370	3333
Speed indicator	198	0	310	22	341	137	180	46	53	24
Altimeter	180	8	112	104	3	159	135	149	26	106
RMI indicator	0	45	20	0	3	6	0	1	0	0
VOR/ILS indicator	1450	962	1433	320	1023	2282	3038	2754	1616	2326
Vertical speed indicator	96	79	116	13	94	423	530	447	29	315
Turn coordinator	0	0	0	0	28	15	0	18	0	0
CDI indicator	1	18	2	0	0	0	0	0	0	0

**Table 7:** Percentage contribution of individual instruments in the process of vision: VFR and IFR pilots.

Instruments	Value (%)									
	VFR Pilot					IFR Pilot				
	P01	P02	P03	P04	P05	P06	P07	P08	P09	P10
Attitude indicator	69.44	32.36	60.94	79.23	67.01	42.46	39.06	39.72	57.89	54.6
Speed indicator	3.14	0.00	6.07	1.00	7.54	2.61	2.82	0.81	1.29	0.39
Altimeter	2.86	0.49	2.19	4.71	0.07	3.03	2.12	2.63	0.64	1.74
RMI indicator	0.00	2.74	0.39	0.00	0.07	0.11	0	0.02	0	0
VOR/ILS indicator	23.02	58.52	28.08	14.48	22.6	43.45	47.68	48.61	39.47	38.11
Vertical speed indicator	1.52	4.81	2.27	0.59	2.08	8.05	8.32	7.89	0.71	5.16
Turn coordinator	0.00	0.00	0.00	0.00	0.62	0.29	0	0.32	0	0
CDI indicator	0.02	1.09	0.04	0.00	0.00	0	0	0	0	0

**Table 8:** Percentage contribution of fixations for the measurements with sharp instrument outlines.

Fixations Registered	Value (%)									
	VFR Pilot					IFR Pilot				
	P01	P02	P03	P04	P05	P06	P07	P08	P09	P10
within the AOI	82.57	23.24	67.80	32.09	65.54	82.02	89.22	79.65	61.57	76.21
outside AOI	17.43	76.76	32.20	67.91	34.46	17.98	10.78	20.35	38.43	23.79

**Table 9:** Percentage contribution of fixations for the measurements with a blurred AOI outline.

Fixations Registered	Value (%)									
	VFR Pilot					IFR Pilot				
	P01	P02	P03	P04	P05	P06	P07	P08	P09	P10
within the AOI	91.80	84.31	87.00	68.43	79.35	89.93	90.57	93.75	90.05	95.19
outside AOI	8.20	15.69	13.00	31.57	20.65	10.07	9.43	6.25	9.95	4.81

**Table 10:** Averaged percentage values of attention using the attention gradation algorithm.

Instruments	VFR Pilot	IFR Pilot
Attitude indicator	52.24	43.10
Speed indicator	5.43	4.47
Altimeter	2.14	4.47
RMI indicator	0.32	0.26
VOR/ILS indicator	35.87	42.24
Vertical speed indicator	3.20	6.01
Turn coordinator	0.65	0.25
CDI indicator	0.16	0.41

**Table 11:** Average percentage contribution of the duration of all fixations depending on the defined area based on the algorithm with instrument outline blur.

Instruments	Value (%)									
	VFR Pilot					IFR Pilot				
	P01	P02	P03	P04	P05	P06	P07	P08	P09	P10
Attitude indicator	64.8	42.9	54.2	43.9	55.4	41.9	42.0	36.8	51.9	42.9
Speed indicator	4.5	3.9	6.6	5.8	6.3	6.4	5.3	4.5	2.3	3.9
Altimeter	3.6	1.9	2.6	1.8	0.7	4.5	3.3	3.5	3.1	1.9
RMI indicator	0.2	0.2	0.4	0.2	0.6	0.5	0.2	0.3	0.1	0.2
VOR/ILS indicator	24.4	45.8	32.3	44.9	31.9	37.8	40.2	46.7	40.6	45.8
Vertical speed indicator	2.4	4.8	3.1	2.8	2.9	8.0	8.5	7.4	1.3	4.8
Turn coordinator	0.1	0.2	0.6	0.4	1.9	0.4	0.1	0.4	0.1	0.2
CDI indicator	0.1	0.4	0.1	0.1	0.2	0.4	0.3	0.4	0.6	0.4

## Abbreviations

The following abbreviations are used in this manuscript:

AGL	Above Ground Level
AMSL	Above Medium Sea Level
AOA	Areas Of Analysis
AOI	Area Of Interest
ATPL(A)	Airline Transport Pilot License
BPS	Bright Pupil System
CAT I ILS	First category of ILS
CDI	Course Deviation Indicator
CPL(A)	Commercial Pilot License
CS	Coil System
DA	Decision Altitude
DH	Decision Height
DPS	Dark Pupil System
EOG	Electrooculography
EPRZ	The code of Jasionka–Rzeszow airport in Poland
HMI	Human Machine Interface
IAS	Indicated Airspeed Indicator
IFR	Instrument Flight Rules
ILS	Instrument Landing System
IMC	Instrument Meteorological Conditions
IR	Instrument Rating
IR marker	Infrared markers
MCSpx	Tobii notation for Media Coordinate System pixels
NM	Nautical Miles
RMI	Radio Magnetic Indicator
RVR	Runway Visual Range
SMI	SensoMotoric Instruments
VFR	Visual Flight Rules
VOR/ILS	Very high-frequency Omnidirectional Range/Instrument Landing System
QNH	Atmospheric Pressure (Q) at Nautical Height
